# Supplementary material for: BRD4-mediated repression of p53 is a target for combination therapy in AML
Source: Nat Commun. 2021 Jan 11;12:241. doi: 10.1038/s41467-020-20378-8 (PMC7801601; doi:10.1038/s41467-020-20378-8)
Supplement: Supplementary file 1 — Supplementary Information [file 41467_2020_20378_MOESM1_ESM.pdf]

Supp Figure 1

A

| | Sample | Cytogenetics | Molecular analysis | TP53 Status |
|---|---|---|---|---|
| 1 | BSMS 71 | 46XY, inv(16) | FLT3-ve, NPM1-ve, CBFB-MYH11 | No mutation detected |
| 2 | BSMS 74 | 46XY | FLT3+ve, NPM1+ve | ND |
| 3 | BSMS 82 | 46XY | FLT3-ve, NPM1-ve | ND |
| 4 | BSMS 84 | 46XY | FLT3-ve, NPM1-ve | ND |
| 5 | BSMS 87 | 46XY, del(5) | FLT3-ve, NPM1-ve | ND |
| 6 | BSMS 103 | 46XY | FLT3+ve, NPM1+ve | No mutation detected |
| 7 | BSMS 104 | 48XY, +19, +21 | FLT3-ve, NPM1-ve | No mutation detected |
| 8 | BSMS 131 | 46XX | FLT3-ve, NPM1-ve | ND |
| 9 | BSMS 136 | 46XX | N/A | No mutation detected |
| 10 | BSMS 256 | 46XY, del(7) | FLT3-ve, NPM1-ve | ND |
| 11 | BSMS 257 | 46XX | FLT3-ve, NPM1-ve, TET2+ve | ND |
| 12 | BSMS 267 | 46XY | FLT3-ve, NPM1+ve | ND |
| 13 | BSMS 269 | 46XY, del (7) | FLT3-ve, NPM1-ve | ND |
| 14 | BSMS 270 | 46XY | N/A | ND |
| 15 | BSMS 273 | 46XY | N/A | ND |

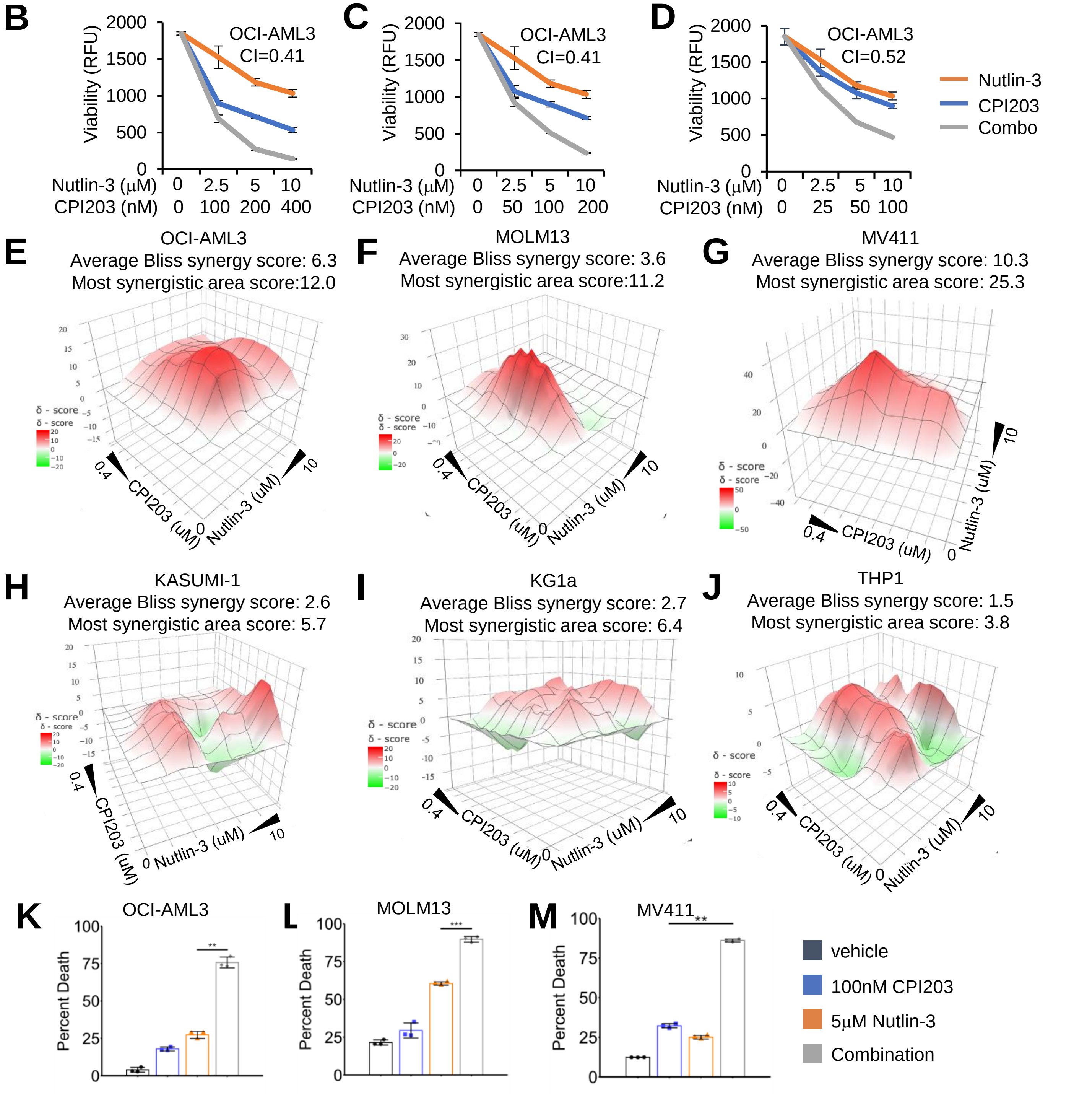

**Supp Figure 1 (continued)**

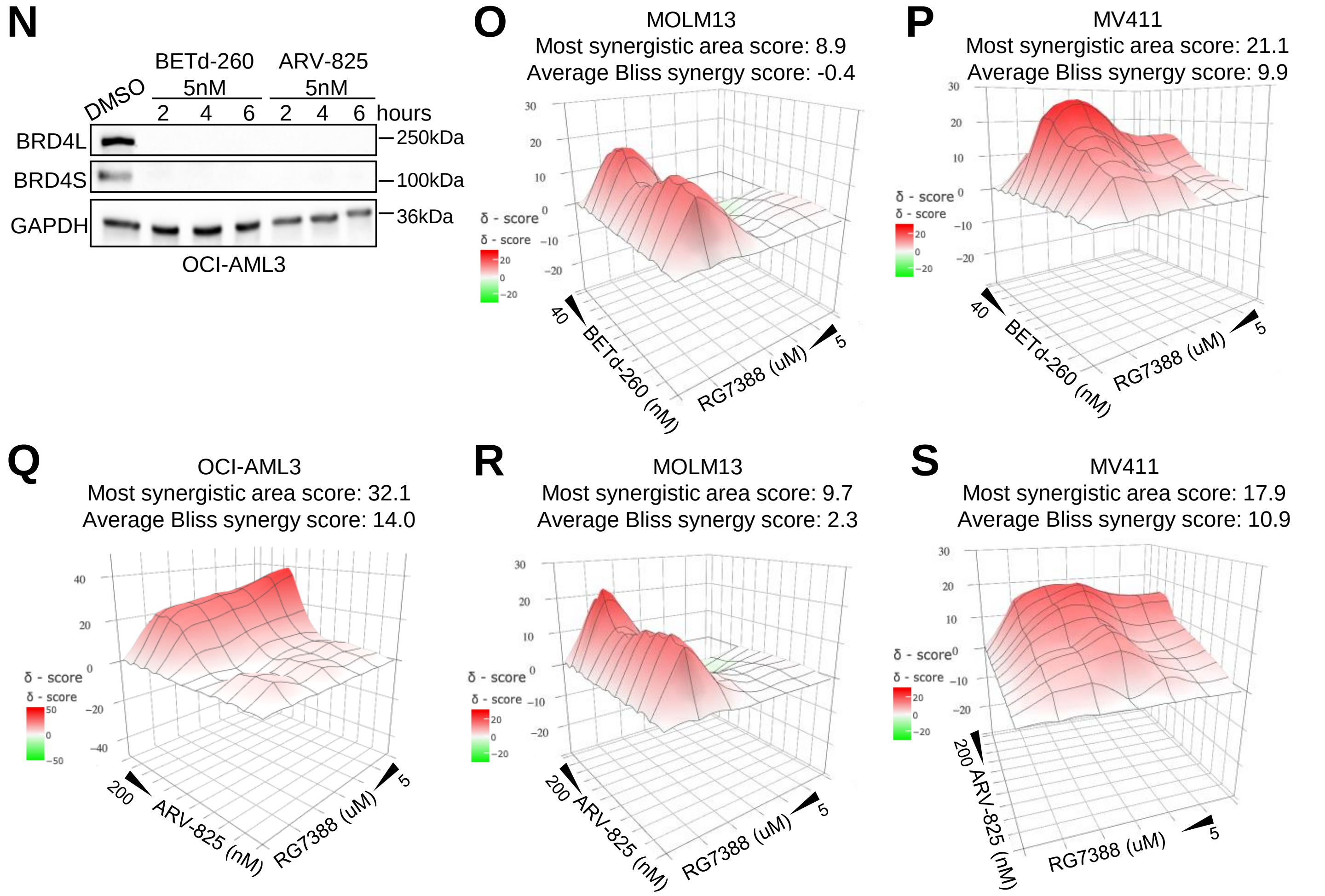

**Supp Figure 1. MDM2 and BET inhibitors are synergistically lethal to primary human AML blasts and AML cell lines with wild-type *TP53*.**

**A.** Summary of the cytogenetic, molecular features and *TP53* status of the 15 primary human AML samples in Figure 1A. FLT3+ve denotes mutation for FLT3 (FLT3-ITD) and FLT3-ve denotes WT. NPM1+ve denotes recurrent AML mutation. NMP1-ve denotes WT. For the 4 indicated samples, all exons of TP53 were sequenced, but no mutations detected. ND: not determined. N/A: not available.

**B-D.** OCI-AML3 cell viability, resazurin assay 72 hours, treatment ratio CPI203:nutlin-3 indicated drug concentrations (Means +/- SD, n=3).

**E-J**. Excess Over Bliss (EOB) plot between Nutlin-3 and CPI203 in indicated cells. Cell viability by CellTiter-Glo, 72 hours. Bliss score, n=4.

**K.** Cell kill in OCI-AML3 cells using annexin-V and propidium iodide, (72hrs) (***=p≤0.001). Percent death = sum of percent single (annexin-V+ or propidium iodide+) and double positive cells (two tailed unpaired t-test, Means +/- SD, n=3).

**L.** Cell kill in the MOLM13 cells using annexin-V and propidium iodide, (72hrs) (***=p≤0.001). Percent death = sum of percent single (annexin-V+ or propidium iodide+) and double positive cells (two tailed unpaired t-test, Means +/- SD, n=3).

**M.** Cell kill in the MV411 cells using annexin-V and propidium iodide, (72hrs) (**=p≤0.01). Percent death = sum of percent single (annexin-V+ or propidium iodide+) and double positive cells (two tailed unpaired t-test, Means +/- SD, n=3).

**N.** Western blot of BRD4 in OCI-AML3 cells treated with BETd-260 or ARV-825 at 5nM.

**O.** EOB plot between RG7388 and BETd-260 in MOLM13 cells. Cell viability by CellTiter-Glo after 24 hours. Bliss score, n=4.

**P.** EOB plot between RG7388 and BETd-260 in MV411 cells. Cell viability by CellTiter-Glo after 24 hours. Bliss score, n=4.

**Q.** EOB plot showing synergistic effects between RG7388 and ARV-825 in OCI-AML3 cells. Cell viability by CellTiter-Glo after 24 hours. Bliss score, n=4.

**R.** EOB plot between RG7388 and ARV-825 in MOLM13 cells. Cell viability by CellTiter-Glo after 24 hours. Bliss score, n=4.

**S.** EOB plot between RG7388 and ARV-825 in MV411 cells. Cell viability by CellTiter-Glo after 24 hours. Bliss score, n=4.

Supp Figure 2

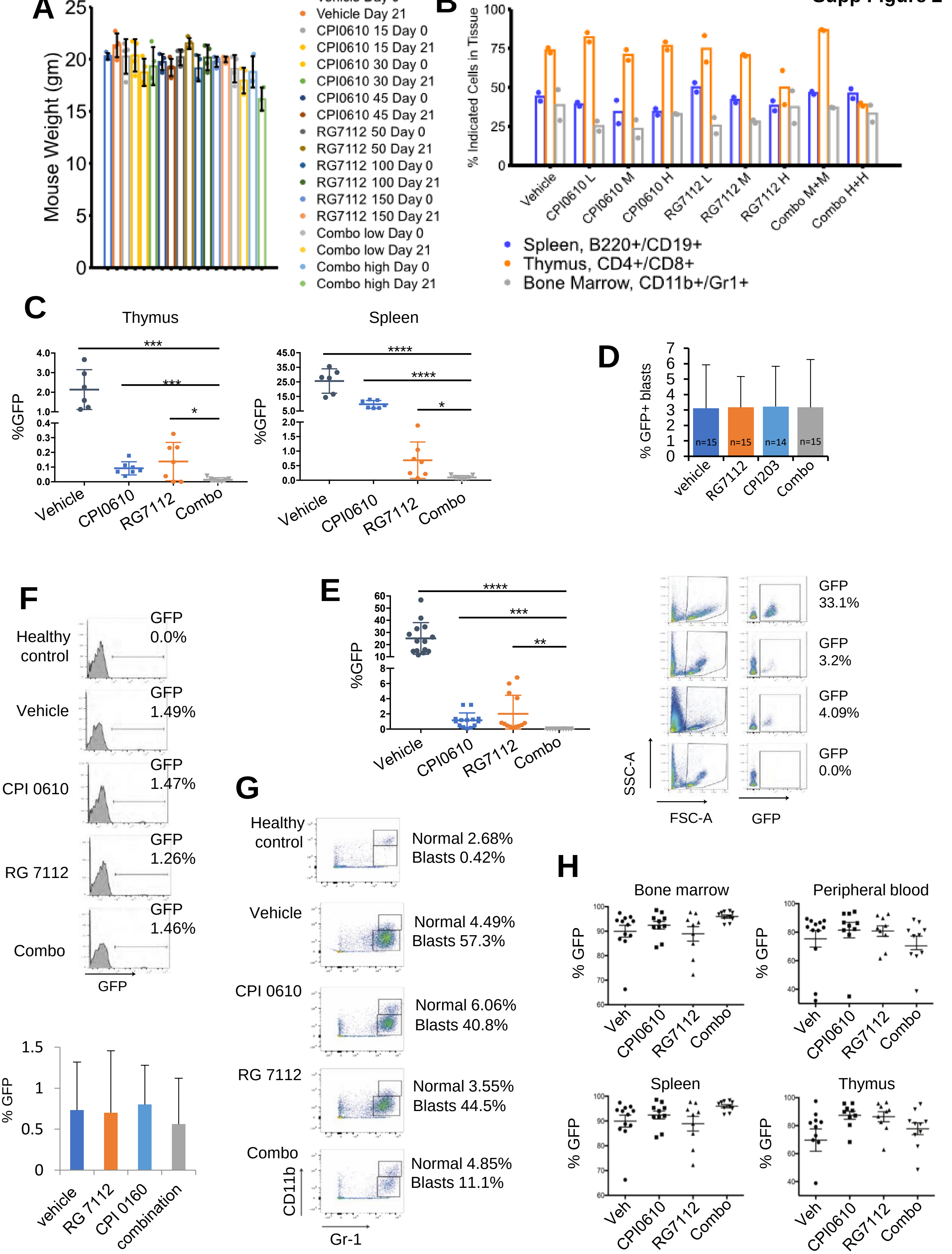

**Supp Figure 2. MDM2 and BET inhibitors cooperate to eradicate AML in *in vivo* mouse models.**
**A**. Normal non-leukemic mouse weights at indicated daily drug doses (mg/kg) at day 0 and on completion of drug treatment (day 21). For combo, low dose = 30mg/kg CPI0610 and 100mg/kg RG7112; high dose = 45mg/kg CPI0610 and 150mg/kg RG7112 (Means +/- SD are shown, n = 4 for all except for combo high day 0  and combo high day 21, where n = 3).
**B**. Percent of indicated cell types in indicated tissue in normal non-leukemic mice treated with vehicle, low, medium or high dose of CPI0610 (15, 30 or 45mg/kg), RG7112 (50, 100, 150mg/kg) or combination (M+M = 30mg/kg CPI0610 and 100mg/kg RG7112; H+H = 45mg/kg CPI0610 and 150mg/kg RG7112).
**C.** Disease burden (percent GFP+ blasts in thymus or spleen) in Trib2 mice after 21 days of drug treatment (****=p≤0.0001, ***=p≤0.001, *=p≤0.05, by two tailed unpaired t-test, Means +/- SD, n = 6 for vehicle, n=7 for single drug and combo conditions).
**D.** Disease burden (percent GFP+ blasts in peripheral blood) in Trib2 mice pre-drug treatment (Error bars are SD, n for each group labeled)
**E.** Disease burden (percent GFP+ blasts in peripheral blood) in Trib2 treated mice after 21 days treatment in peripheral blood according to treatment condition. Right panel shows representative FACS data (****=p≤0.0001, ***=p≤0.001, **=p≤0.01, two tailed unpaired t-test, Means +/- SD, n=14, gating strategy for sorting GFP Positive cells was live cells>cell profile>single cells>GFP positive cells).
**F.** MLL-AF9 disease burden (percent GFP+ blasts in peripheral blood) pre-drug treatment. Top shows representative FACS data (Gating strategy for sorting GFP Positive cells was live cells>cell profile>single cells>GFP positive cells.).
**G.** Representative MLL-AF9 disease burden in peripheral blood, based on percent $CD11b^{low}$ Gr1-expressing immature blasts known to expand in AML (Keeshan et al. 2006), after 7 days of drug treatment. Also shown are the more normal, mature myeloid cells ($CD11b^{high}$ Gr1+) (Gating strategy for sorting GFP Positive cells was live cells>cell profile>single cells>GFP positive cells).
**H.** % MLL-AF9 GFP+ blasts at time of cull in indicated tissues after indicated drug treatments.

## Supp Figure 3

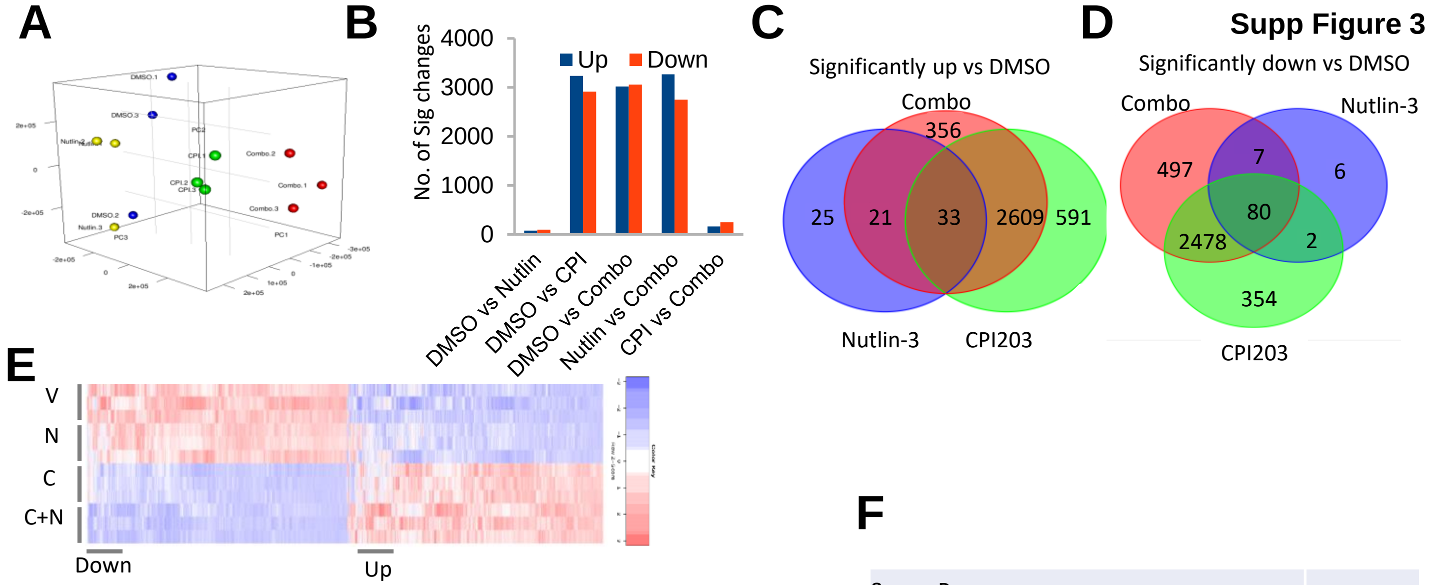


F

| Synergy Down genes | |
|---|---|
| **Ingenuity Canonical Pathways** | -log(p-value) |
| Mitotic Roles of Polo-Like Kinase | 3.74 |
| Cell Cycle: G2/M DNA Damage Checkpoint Regulation | 2.92 |
| Hereditary Breast Cancer Signaling | 2.36 |
| Myc Mediated Apoptosis Signaling | 2.32 |
| DNA Methylation and Transcriptional Repression Signaling | 2.04 |
| 3-phosphoinositide Biosynthesis | 1.94 |
| 5-aminoimidazole Ribonucleotide Biosynthesis I | 1.9 |
| Coenzyme A Biosynthesis | 1.9 |
| Diphthamide Biosynthesis | 1.9 |
| Pyrimidine Ribonucleotides Interconversion | 1.82 |
| **Synergy Up genes** | |
| **Ingenuity Canonical Pathways** | -log(p-value) |
| p53 Signaling | 8.33 |
| SAPK/JNK Signaling | 3.53 |
| Autophagy | 3.25 |
| IL-8 Signaling | 3.17 |
| Agrin Interactions at Neuromuscular Junction | 2.76 |
| Molecular Mechanisms of Cancer | 2.74 |
| NRF2-mediated Oxidative Stress Response | 2.67 |
| Pancreatic Adenocarcinoma Signaling | 2.59 |
| HMGB1 Signaling | 2.39 |
| TNFR2 Signaling | 2.36 |

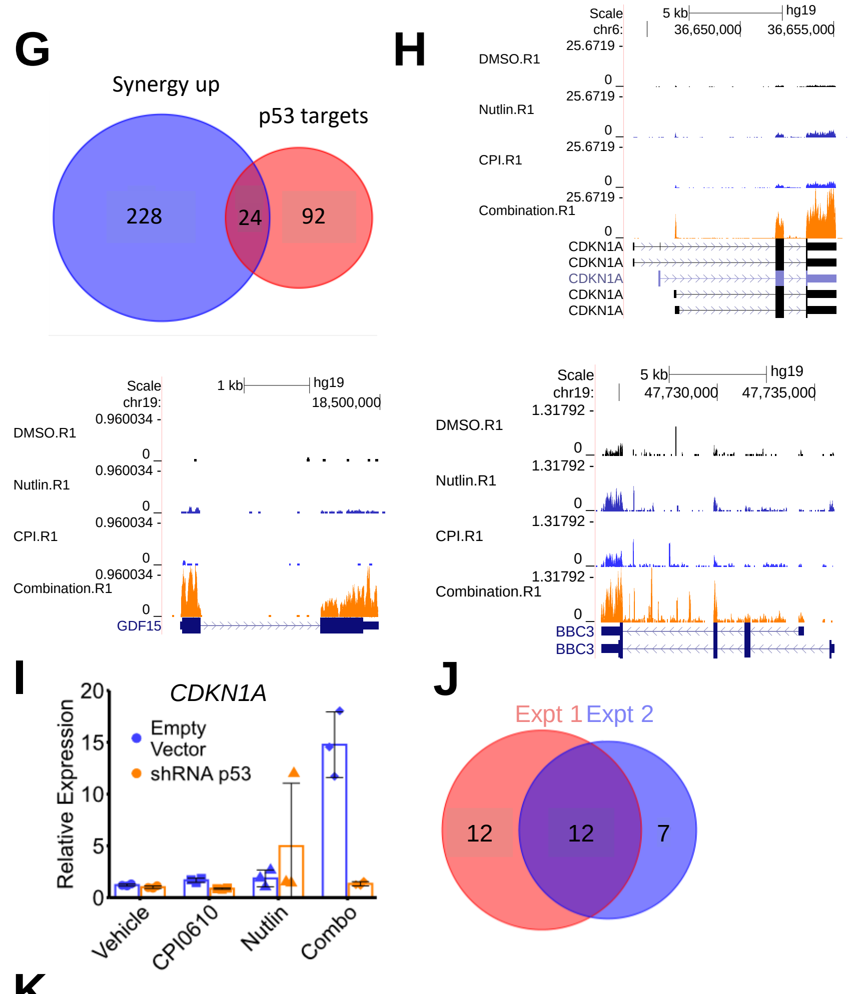


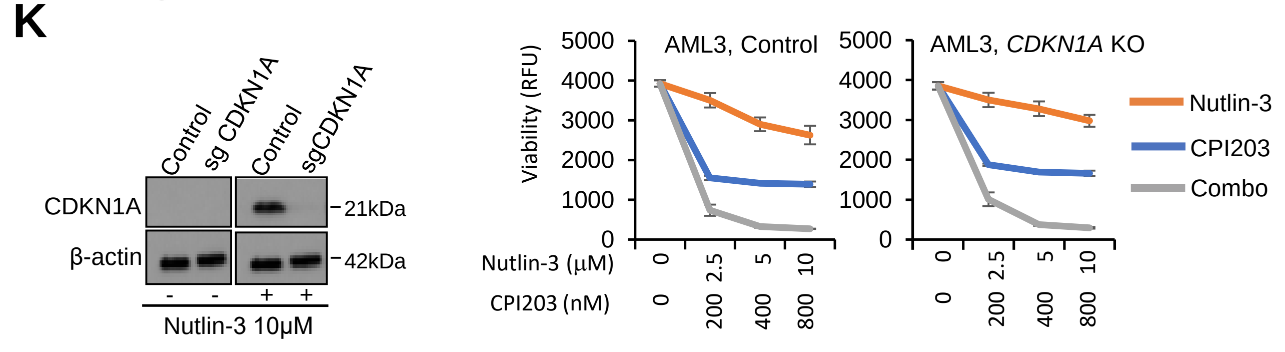

**Supp Figure 3. BET inhibitors potentiate activation of p53 target genes by p53.**
**A.** A principal component analysis of RNA-seq data of the OCI-AML3 cell line (from Figure 3E), according to treatment condition.
**B.** Number of significant gene expression changes between treatment conditions in RNA-seq data of treated OCI-AML3 cells .
**C.** Venn diagram of genes significantly up-regulated in RNA-seq data, according to treatment condition of OCI-AML3 cells.
**D.** Venn diagram of genes significantly down-regulated in RNA-seq data, according to treatment condition of OCI-AML3 cells.
**E.** Heat map of all significant changes in gene expression in RNA-seq data of OCI-AML3 cells according to treatment condition.
**F.** An IPA analysis of synergistically up-regulated and down-regulated genes (synergy as defined in Figure 3E, p-values from fisher's exact test).
**G.** Venn diagram showing the number of high-confidence p53 target genes synergistically up-regulated by the drug combination**.**
**H.** Representative examples of RNA-seq gene tracks for the p53 target genes *CDKN1A*, *GDF15* and *BBC3*, according to treatment condition.
**I.** qPCR assessment of expression of CDKN1A in control (empty-vector) OCI-AML3 cells and shRNA p53 OCI-AML3 cells, according to treatment condition (Means +/- SD are shown, n=3)**.**
**J.** Venn diagram showing number of expression changes in p53 target genes in OCI-AML3 cells expressing wild-type p53 in the first and second RNA-seq experiments (i.e. Figures 3F and 3N, respectively).
**K.** Western blot analysis of control (empty-vector) OCI-AML3 cells and CRISPR/CAS9 CDKN1A knock out OCI-AML3 cells, treated with vehicle or 10μM nutlin-3 (left); and a resazurin analysis of control (empty-vector) OCI-AML3 cells and CDKN1A knock out OCI-AML3 cells according to treatment condition, following 72 hours of drug treatment (right) (Means +/- SD are shown, n=3).

**Supp Figure 4**

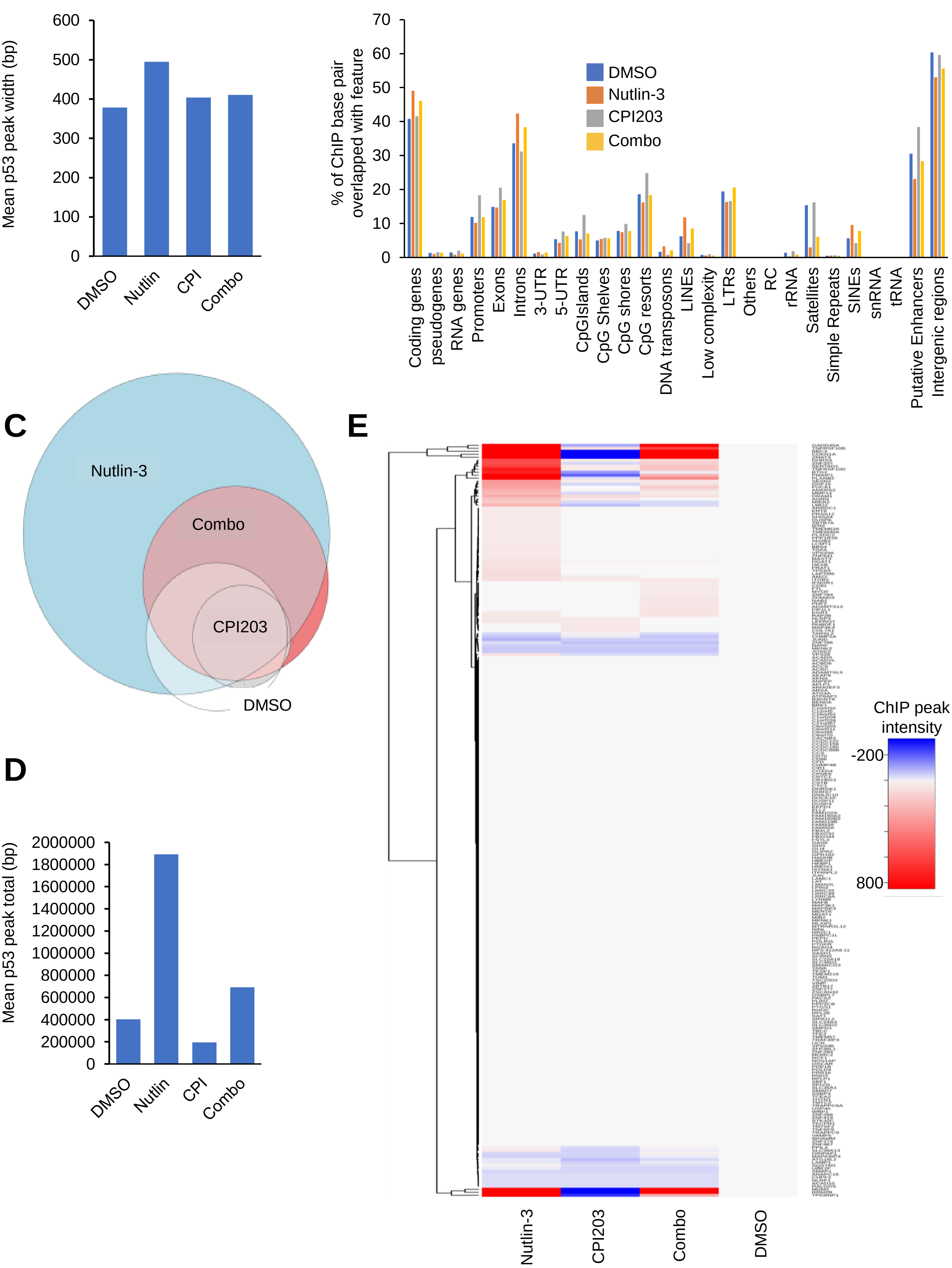

**Supp Figure 4. BET inhibitors do not stabilize p53 target mRNAs nor increase binding of p53 to target genes.**

**A.** Mean p53 peak width in base pairs (peaks present in at least 2 out of 3 ChIP-seq replicates; mean of 2-3 replicates) after the indicated drug treatments of OCI-AML3 cells.

**B.** Genomic distribution of p53 binding sites identified by ChIP-seq (peaks present in at least 2 out of 3 replicates) after the indicated drug treatments of OCI-AML3 cells.

**C.** Venn diagram showing relative overlap of p53 ChIP-seq peaks (peaks present in at least 2 out of 3 replicates) after the indicated drug treatments of OCI-AML3 cells.

**D.** Mean total base pairs covered by p53 ChIP-seq peaks (peaks present in at least 2 out of 3 replicates; mean of 2-3 replicates) after the indicated drug treatments of OCI-AML3 cells.

**E.** Heat map of p53 ChIP-seq peaks (peaks present in at least 2 out of 3 replicates) at 252 synergy up genes (rows) after the indicated drug treatments (columns) of OCI-AML3 cells.

**Supp Figure 5**

**A**

| Rank | Upstream Regulator | P-value of overlap | Rank | Upstream Regulator | P-value of overlap |
|---|---|---|---|---|---|
| 1 | E2F4 | 1.13E-24 | 6 | FOXM1 | 2.02E-12 |
| 2 | NUPR1 | 1.17E-23 | 7 | CCND1 | 6.38E-11 |
| 3 | MYC | 4.16E-21 | 8 | SPI1 | 7.13E-10 |
| 4 | p53 | 9.60E-19 | 9 | TCF4 | 9.01E-10 |
| 5 | E2F1 | 2.20E-17 | 10 | TP63 | 4.36E-09 |

**B**

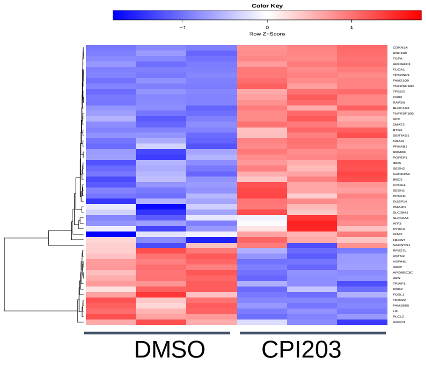


**C**

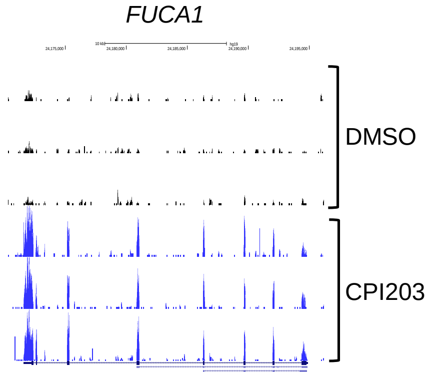


**D**

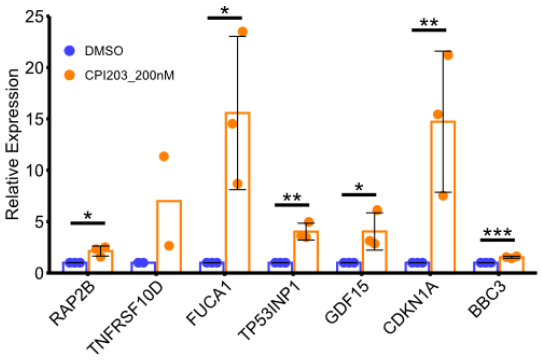


**E**

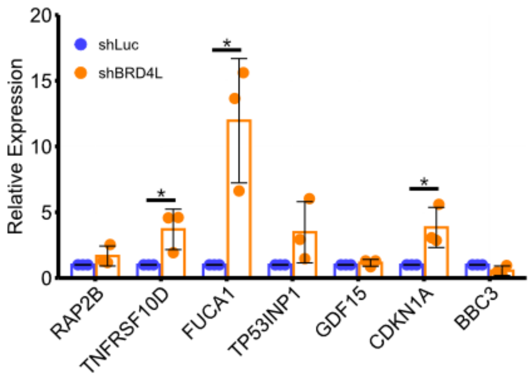


**F**

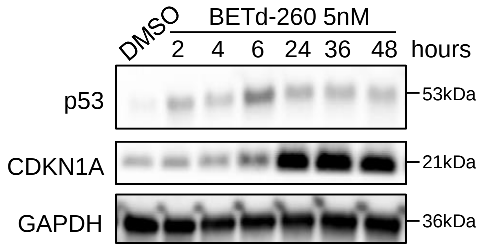

**Supp Figure 5. Inhibition of BET family proteins activates p53.**

**A.** Ingenuity pathway analysis (IPA) identifies p53 as a candidate regulator of genes differentially expressed between control and CPI203-treated AML3 cells. p53 is predicted to be activated, with an activation z-score 5.802 (fisher's exact test).

**B.** Heat map of all significant changes in gene expression of known p53 target genes (from Ref. 34) in RNA-seq data of OCI-AML3 cells, control (DMSO) vs CPI203 treated AML3 cells (3 replicates of each).

**C.** RNA-seq sequence tags from control (DMSO) and CPI203-treated AML3 cells aligned to *FUCA1* gene (3 replicates of each).

**D.** qPCR analysis of indicated p53 target genes in samples from Figure 3E (***=p≤0.001, **=p≤0.01, *=p≤0.05, two tailed unpaired t-test, Means +/- SD are shown, n=3).

**E.** qPCR analysis of indicated p53 target genes in OCI-AML3 cells expressing small hairpin RNA(shRNA) specific for luciferase gene (shLuc, control) or BRD4L (shBRD4L) (*=p≤0.05, two tailed unpaired t-test, Means +/- SD are shown, n=3).

**F.** Western blot of p53 and CDKN1A in OCI-AML3 cells treated with BETd-260 at 5nM for different times.

**Supp Figure 6**

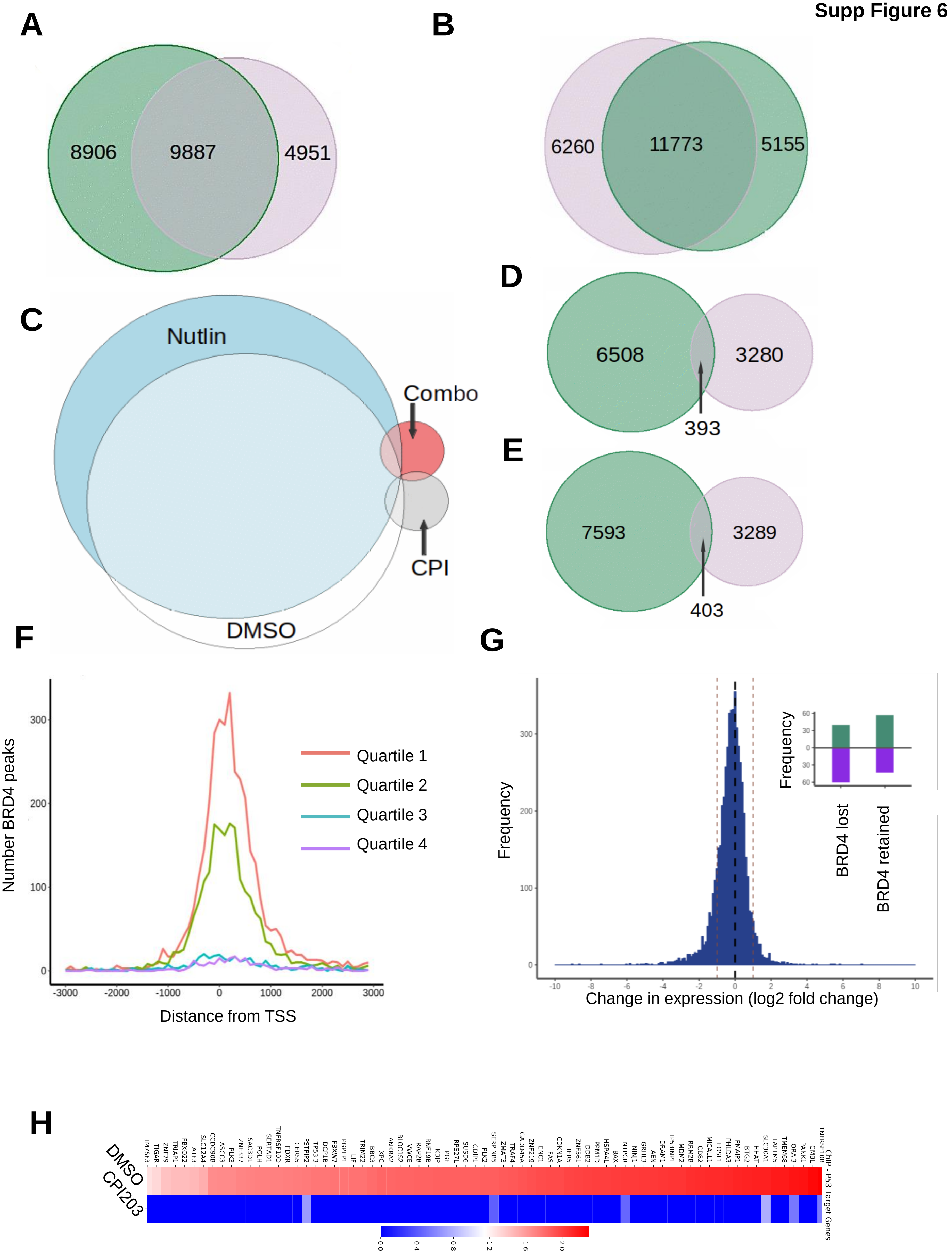

**Supp Figure 6. BRD4 ChIP-seq data**

**A.** Venn diagram showing overlap of 2 replicates of BRD4 ChIP-seq peaks in OCI-AML3 cells treated with DMSO

**B.** Venn diagram showing overlap of 2 replicates of BRD4 ChIP-seq peaks in OCI-AML3 cells treated with nutlin-3

**C.** Venn diagram showing overlap of BRD4 ChIP-seq peaks (peaks present in 2 out of 2 replicates) in OCI-AML3 cells treated with DMSO, nutlin-3, CPI203 or combo.

**D.** Venn diagram showing overlap of 2 replicates of BRD4 ChIP-seq peaks in OCI-AML3 cells treated with CPI203

**E.** Venn diagram showing overlap of 2 replicates of BRD4 ChIP-seq peaks in OCI-AML3 cells treated with CPI203 and nutlin-3.

**F.** Histogram of number of BRD4 ChIP-seqs centered around gene TSS (0) for genes in the highest (quartile 1) to lowest (quartile 4) quartiles of expression in DMSO treated OCI-AML3 cells.

**G.** The histogram shows the spread of change in expression for genes losing BRD4 binding after treatment with CPI203. Loss of BRD4 on CPI203 treatment was associated with a decrease in gene expression ($p<0.05$). Inset figure: BRD4 peaks overlapping TSS were divided into two groups, those losing BRD4 on CPI203 treatment and those retaining BRD4. Plotted is the % change in expression after CPI203 treatment for the genes within each group.

**H**. Heat map of BRD4 ChIP-seq peaks in known p53 target genes from Control (DMSO) and CPI203 treatment. Values represent the aggregated read counts associated with each gene across samples in log scale.

**Supp Figure 7**

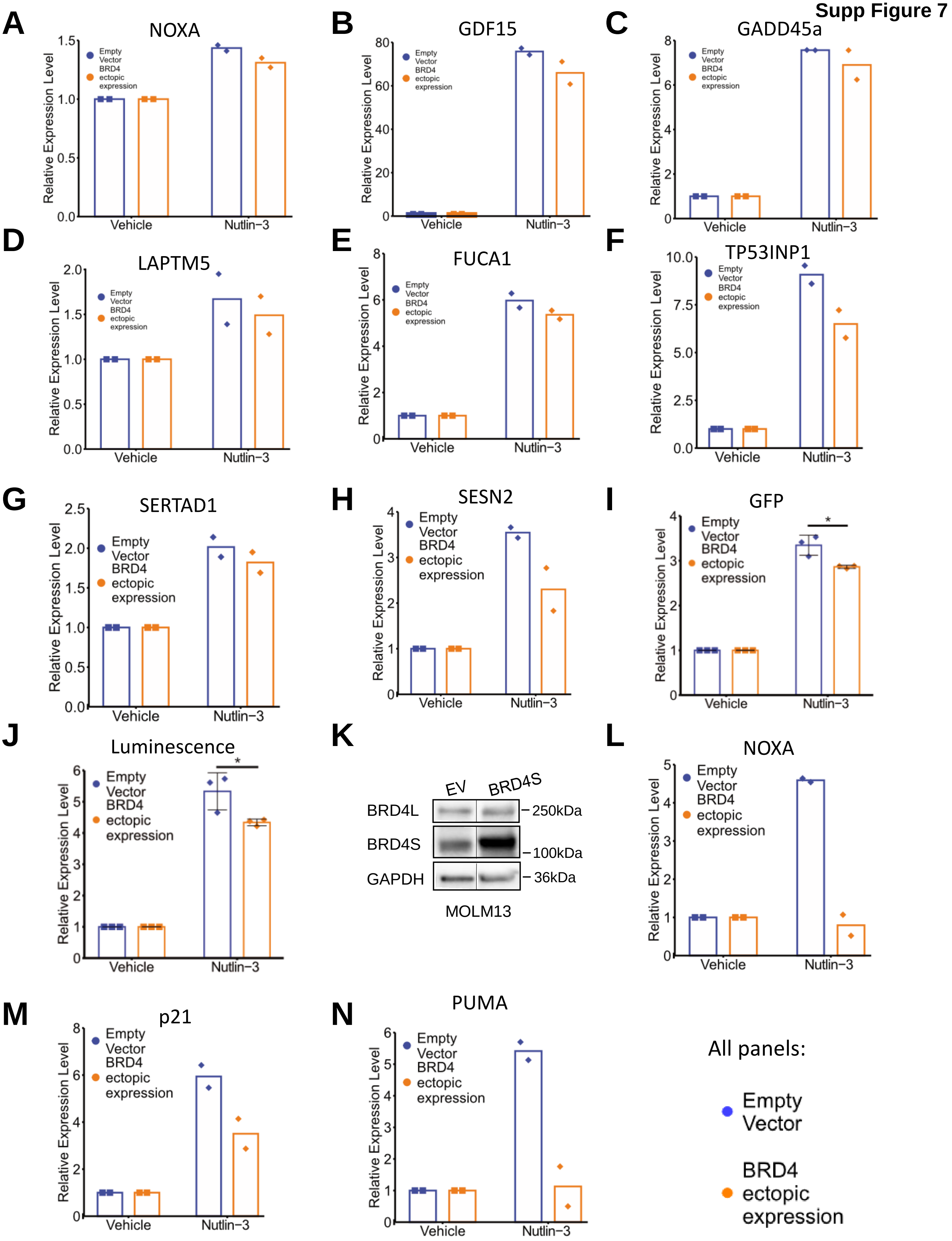

**Supp Figure 7. BRD4 represses p53 target genes.**
**A.** qPCR analysis of *NOXA* in OCI-AML3 cells ectopically expressing BRD4S, in absence or presence of nutlin-3 (2 replicates of each).
**B.** qPCR analysis of *GDF15* in OCI-AML3 cells ectopically expressing BRD4S, in absence or presence of nutlin-3 (2 replicates of each).
**C.** qPCR analysis of *GADD45a* in OCI-AML3 cells ectopically expressing BRD4S, in absence or presence of nutlin-3 (2 replicates of each).
**D.** qPCR analysis of *LAPTM5* in OCI-AML3 cells ectopically expressing BRD4S, in absence or presence of nutlin-3 (2 replicates of each).
**E.** qPCR analysis of *FUCA1* in OCI-AML3 cells ectopically expressing BRD4S, in absence or presence of nutlin-3 (2 replicates of each).
**F.** qPCR analysis of *TP53INP1* in OCI-AML3 cells ectopically expressing BRD4S, in absence or presence of nutlin-3 (2 replicates of each).
**G.** qPCR analysis of *SERTAD1* in OCI-AML3 cells ectopically expressing BRD4S, in absence or presence of nutlin-3 (2 replicates of each).
**H.** qPCR analysis of *SESN2* in OCI-AML3 cells ectopically expressing BRD4S, in absence or presence of nutlin-3 (2 replicates of each).
**I.** GFP measurement in OCI-AML3 cells ectopically expressing BRD4S, in absence or presence of nutlin-3(*=$p \leq 0.05$ by a two-tailed unpaired t-test, n=3).
**J.** Luminescence measurement in OCI-AML3 cells ectopically expressing BRD4S, in absence or presence of nutlin-3(*=$p \leq 0.05$ by a two-tailed unpaired t-test, n=3).
**K.** Western blot for BRD4 in MOLM13 cells ectopically expressing the short isoform of BRD4 (BRD4S). BRD4L is the long isoform.
**L.** qPCR analysis of *NOXA* in OCI-AML3 cells ectopically expressing BRD4S, in absence or presence of nutlin-3 (**=$p \leq 0.01$) (2 replicates of each).
**M.** qPCR analysis of *CDKN1A* in OCI-AML3 cells ectopically expressing BRD4S, in absence or presence of nutlin-3 (2 replicates of each).
**N.** qPCR analysis of *PUMA* in OCI-AML3 cells ectopically expressing BRD4S, in absence or presence of nutlin-3 (*=$p \leq 0.05$) (2 replicates of each).
