# Supplementary material for: BRD4-mediated repression of p53 is a target for combination therapy in AML
Source: Nat Commun. 2021 Jan 11;12:241. doi: 10.1038/s41467-020-20378-8 (PMC7801601; doi:10.1038/s41467-020-20378-8)
Supplement: Supplementary file 3 — Description of Additional Supplementary Files [file 41467_2020_20378_MOESM3_ESM.pdf]

Description of Additional Supplementary Files

Supplementary Data 1:
List of genes that are synergistically up-regulated with Nutlin-3 and CPI203 combination treatment in OCI-AML3 cells.

Supplementary Data 2:
List of genes that are synergistically down-regulated with Nutlin-3 and CPI203 combination treatment in OCI-AML3 cells.

Supplementary Data 3:
List of genes whose synergistic up-regulation with Nutlin-3 and CPI203 combination treatment, is dependent on p53 in OCI-AML3 cells.

Supplementary Data 4:
List of PCR primers used for qPCR.

Supplementary Data 5:
List of plasmids used and where they were from.

Supplementary Data 6:
List of antibodies and dilutions used in western blots.

Supplementary Data 7:
Accession numbers and tokens for accessing RNA-seq and ChIP-seq datasets.

Supplementary Data 8:
List of Patient information and relevant covariate data.
