# Supplementary material for: BRD4-mediated repression of p53 is a target for combination therapy in AML
Source: Nat Commun. 2021 Jan 11;12:241. doi: 10.1038/s41467-020-20378-8 (PMC7801601; doi:10.1038/s41467-020-20378-8)
Supplement: Supplementary file 5 — Reporting Summary [file 41467_2020_20378_MOESM5_ESM.pdf]

nature research

Corresponding author(s): Anne-Louise Latif, Ashley Newcombe, Sha Li, Kathryn Gilroy, Neil Robertson, Xue Lei, Helen Stewart, John Cole, Maria Terradas Terradas, Loveena Rishi, Lynn McGarry, Claire McKeeve, Claire
Reid, William Clark, Joana Campos, Kristina Kirschner, Jonathan Lopez, Jun-Ichi Sakamaki, Jennifer
Morton, Kevin M. Ryan, Stephen Tait, Sheela Abraham, Tessa Holyoake, Brian Higgins, Xu Huang, Karen Blyth, Mhairi Copland, Tim Chevassut, Karen Keeshan, Peter D. Adams

Last updated by author(s): Nov 20, 2020

# Reporting Summary

Nature Research wishes to improve the reproducibility of the work that we publish. This form provides structure for consistency and transparency in reporting. For further information on Nature Research policies, see our Editorial Policies and the Editorial Policy Checklist.

## Statistics

For all statistical analyses, confirm that the following items are present in the figure legend, table legend, main text, or Methods section.

| n/a | Confirmed | |
|---|---|---|
| ☐ | ☒ | The exact sample size (*n*) for each experimental group/condition, given as a discrete number and unit of measurement |
| ☐ | ☒ | A statement on whether measurements were taken from distinct samples or whether the same sample was measured repeatedly |
| ☐ | ☒ | The statistical test(s) used AND whether they are one- or two-sided<br>*Only common tests should be described solely by name; describe more complex techniques in the Methods section.* |
| ☐ | ☒ | A description of all covariates tested |
| ☐ | ☐ | A description of any assumptions or corrections, such as tests of normality and adjustment for multiple comparisons |
| ☐ | ☐ | A full description of the statistical parameters including central tendency (e.g. means) or other basic estimates (e.g. regression coefficient) AND variation (e.g. standard deviation) or associated estimates of uncertainty (e.g. confidence intervals) |
| ☐ | ☒ | For null hypothesis testing, the test statistic (e.g. *F*, *t*, *r*) with confidence intervals, effect sizes, degrees of freedom and *P* value noted<br>*Give P values as exact values whenever suitable.* |
| ☐ | ☐ | For Bayesian analysis, information on the choice of priors and Markov chain Monte Carlo settings |
| ☐ | ☐ | For hierarchical and complex designs, identification of the appropriate level for tests and full reporting of outcomes |
| ☐ | ☐ | Estimates of effect sizes (e.g. Cohen's *d*, Pearson's *r*), indicating how they were calculated |

*Our web collection on statistics for biologists contains articles on many of the points above.*

## Software and code

Policy information about availability of computer code

| | |
|---|---|
| Data collection | All Computer Code Calculations were done with the commercially available software products described in the methods sections (e.g. Bliss, DESeq2, etc.) |
| Data analysis | All Computer Code Calculations were done with the commercially available software products described in the methods sections (e.g. Bliss, DESeq2, etc.) |

For manuscripts utilizing custom algorithms or software that are central to the research but not yet described in published literature, software must be made available to editors and reviewers. We strongly encourage code deposition in a community repository (e.g. GitHub). See the Nature Research guidelines for submitting code & software for further information.

## Data

Policy information about availability of data

All manuscripts must include a data availability statement. This statement should provide the following information, where applicable:

- Accession codes, unique identifiers, or web links for publicly available datasets
- A list of figures that have associated raw data
- A description of any restrictions on data availability

Chip Seq data has been deposited in the Gene Expression Omnibus (GEO) under accession codes GSE132246 and GSE132247. RNA seq data has been deposited in the Gene Exression Omnibus (GEO) under accession codes GSE132244 and GSE132245. All other data supporting supporting the findings of this study are available from the corresponding author upon reasonable request.

RNA-seq
https://www.ncbi.nlm.nih.gov/geo/query/acc.cgi?acc=GSE132244
reviewer token: qtojkkwkvpqtxyb

RNA-seq
https://www.ncbi.nlm.nih.gov/geo/query/acc.cgi?acc=GSE132245
reviewer token: mdulsccgdnsptep

ChIP-seq
https://www.ncbi.nlm.nih.gov/geo/query/acc.cgi?acc=GSE132246
reviewer token: wdqrgaiohjgvvgx

ChIP-seq
https://www.ncbi.nlm.nih.gov/geo/query/acc.cgi?acc=GSE132247
reviewer token: qbevckewvpgdfmz

# Field-specific reporting

Please select the one below that is the best fit for your research. If you are not sure, read the appropriate sections before making your selection.

☒ Life sciences ☐ Behavioural & social sciences ☐ Ecological, evolutionary & environmental sciences

For a reference copy of the document with all sections, see nature.com/documents/nr-reporting-summary-flat.pdf

# Life sciences study design

All studies must disclose on these points even when the disclosure is negative.

| | |
|---|---|
| Sample size | Sample sizes were determined based on previous experiments with the cell lines and animals |
| Data exclusions | No data was excluded from the analyses |
| Replication | All attempts at replication have been successful. |
| Randomization | Allocation of samples into groups was done at random |
| Blinding | *Describe whether the investigators were blinded to group allocation during data collection and/or analysis. If blinding was not possible, describe why OR explain why blinding was not relevant to your study.* |

# Reporting for specific materials, systems and methods

We require information from authors about some types of materials, experimental systems and methods used in many studies. Here, indicate whether each material, system or method listed is relevant to your study. If you are not sure if a list item applies to your research, read the appropriate section before selecting a response.

## Materials & experimental systems

| n/a | Involved in the study | |
|---|---|---|
| ☐ | ☒ | Antibodies |
| ☐ | ☒ | Eukaryotic cell lines |
| ☐ | ☐ | Palaeontology and archaeology |
| ☐ | ☒ | Animals and other organisms |
| ☐ | ☒ | Human research participants |
| ☐ | ☐ | Clinical data |
| ☐ | ☐ | Dual use research of concern |

## Methods

| n/a | Involved in the study | |
|---|---|---|
| ☐ | ☒ | ChIP-seq |
| ☐ | ☒ | Flow cytometry |
| ☐ | ☐ | MRI-based neuroimaging |

# Antibodies

| | |
|---|---|
| Antibodies used | Sigma, A1978, RRID:AB_476692 (Actin); Santa Cruz, 397, RRID:AB_632126 (p21/CDKN1A); Santa Cruz, 764, RRID:AB_631276 (MYC); Santa Cruz, 965, RRID:AB_627920 (MDM2); Cell Signalling, 9542, RRID:AB_2160739 (PARP); Imgenex, 458, RRID:AB_1151450 (IgG); Santa Cruz, DO1, 126, 30 RRID:AB_628082 (TP53); Abcam, 13654, RRID:AB_300536 (NOXA); Abcam, 128874, RRID:AB_11145462 (BRD4); Cell signaling, 2870, RRID:AB_2290370 (BCL2) |
| Validation | Antibody validation in the lab was done by knockout, or by previous work with the antibody. |

# Eukaryotic cell lines

Policy information about cell lines

| | |
|---|---|
| Cell line source(s) | THP1 was obtained from ATCC, KASUMI-1, MOLM-13 and OCI-AML3 from DSMZ, KG1a and MV411 were gifted from Professor Mhairi Copland and Dr. Xu Huang, respectively (both Paul O'Gorman Leukemia Research Centre, Glasgow). |
| Authentication | Authenticity of all lines was confirmed by genotyping. |
| Mycoplasma contamination | Lines were subjected to regular mycoplasma testing and any lines positive for mycoplasma were not used in experiments. |
| Commonly misidentified lines (See ICLAC register) | OC-AML-3 is listed in the ICLAC register as being contaminated with OC-AML-2. OC-AML-2 is a p53-wt AML line, which, for the purpose of this study, is functionally identical to OC-AML-3. |

# Palaeontology and Archaeology

| | |
|---|---|
| Specimen provenance | *Provide provenance information for specimens and describe permits that were obtained for the work (including the name of the issuing authority, the date of issue, and any identifying information).* |
| Specimen deposition | *Indicate where the specimens have been deposited to permit free access by other researchers.* |
| Dating methods | *If new dates are provided, describe how they were obtained (e.g. collection, storage, sample pretreatment and measurement), where they were obtained (i.e. lab name), the calibration program and the protocol for quality assurance OR state that no new dates are provided.* |

☐ Tick this box to confirm that the raw and calibrated dates are available in the paper or in Supplementary Information.

| | |
|---|---|
| Ethics oversight | *Identify the organization(s) that approved or provided guidance on the study protocol, OR state that no ethical approval or guidance was required and explain why not.* |

Note that full information on the approval of the study protocol must also be provided in the manuscript.

# Animals and other organisms

Policy information about studies involving animals; ARRIVE guidelines recommended for reporting animal research

| | |
|---|---|
| Laboratory animals | mus musculus, c57bl\6, male, 4 months |
| Wild animals | *Provide details on animals observed in or captured in the field; report species, sex and age where possible. Describe how animals were caught and transported and what happened to captive animals after the study (if killed, explain why and describe method; if released, say where and when) OR state that the study did not involve wild animals.* |
| Field-collected samples | *For laboratory work with field-collected samples, describe all relevant parameters such as housing, maintenance, temperature, photoperiod and end-of-experiment protocol OR state that the study did not involve samples collected from the field.* |
| Ethics oversight | *Identify the organization(s) that approved or provided guidance on the study protocol, OR state that no ethical approval or guidance was required and explain why not.* |

Note that full information on the approval of the study protocol must also be provided in the manuscript.

# Human research participants

Policy information about studies involving human research participants

| | |
|---|---|
| Population characteristics | Relevant co-variate data on patients can be found in supplemental data 8. |
| Recruitment | All primary bone marrow aspirates were taken from routine diagnostic specimens after informed consent of the patients. |
| Ethics oversight | The project received approval from the local ethics committee (Brighton and Sussex University Hospitals NHS Trust Research and Development Committee) as The Brighton Blood Disorder Study, references 09/025/CHE and 09/H1107/1) and was conducted in accordance with the Declaration of Helsinki. |

Note that full information on the approval of the study protocol must also be provided in the manuscript.

# Clinical data

Policy information about clinical studies

All manuscripts should comply with the ICMJEguidelines for publication of clinical research and a completedCONSORT checklist must be included with all submissions.

| | |
|---|---|
| Clinical trial registration | *Provide the trial registration number from ClinicalTrials.gov or an equivalent agency.* |
| Study protocol | *Note where the full trial protocol can be accessed OR if not available, explain why.* |
| Data collection | *Describe the settings and locales of data collection, noting the time periods of recruitment and data collection.* |
| Outcomes | *Describe how you pre-defined primary and secondary outcome measures and how you assessed these measures.* |

# Dual use research of concern

Policy information about dual use research of concern

## Hazards

Could the accidental, deliberate or reckless misuse of agents or technologies generated in the work, or the application of information presented in the manuscript, pose a threat to:

| No | Yes | |
|---|---|---|
| ☒ | ☐ | Public health |
| ☒ | ☐ | National security |
| ☒ | ☐ | Crops and/or livestock |
| ☒ | ☐ | Ecosystems |
| ☒ | ☐ | Any other significant area |

## Experiments of concern

Does the work involve any of these experiments of concern:

| No | Yes | |
|---|---|---|
| ☒ | ☐ | Demonstrate how to render a vaccine ineffective |
| ☒ | ☐ | Confer resistance to therapeutically useful antibiotics or antiviral agents |
| ☒ | ☐ | Enhance the virulence of a pathogen or render a nonpathogen virulent |
| ☒ | ☐ | Increase transmissibility of a pathogen |
| ☒ | ☐ | Alter the host range of a pathogen |
| ☒ | ☐ | Enable evasion of diagnostic/detection modalities |
| ☒ | ☐ | Enable the weaponization of a biological agent or toxin |
| ☒ | ☐ | Any other potentially harmful combination of experiments and agents |

# ChIP-seq

## Data deposition

☒ Confirm that both raw and final processed data have been deposited in a public database such as GEO.

☒ Confirm that you have deposited or provided access to graph files (e.g. BED files) for the called peaks.

Data access links
*May remain private before publication.*

ChIP-seq
https://www.ncbi.nlm.nih.gov/geo/query/acc.cgi?acc=GSE132246
reviewer token: wdqrgaiohjgvvgx

ChIP-seq
https://www.ncbi.nlm.nih.gov/geo/query/acc.cgi?acc=GSE132247
reviewer token: qbevckewvpgdfmz

Files in database submission

GSE132246_RAW.tar
GSE132247_RAW.tar

Genome browser session
(e.g. UCSC)

*Provide a link to an anonymized genome browser session for "Initial submission" and "Revised version" documents only, to enable peer review. Write "no longer applicable" for "Final submission" documents.*

## Methodology

Replicates

3 replicates per condition; robust peaks were called as 2 of the 3 samples having a peak at the same location.

Sequencing depth

Sequencing depth is 30Gb according to manufacturer's guidelines for the the High Output Kit V2 with 75 cycles on the Illumina NextSeq500

Antibodies

BRD4 antibody (Abcam, 128874), p53 antibody (Santa Cruz, DO1), (rabbit
or mouse IgG was used as a negative control (Sigma, M7023))

Peak calling parameters

Regions of BRD4 occupancy were determined using the SICER algorithm with a redundancy threshold of 1, a window size of 200, a fragment size of 150, an effective genome fraction of 0.75, a gap size of 200 and an FDR corrected p-value of 0.01 (suited to broader BRD4 peaks). To reduce experimental bias, a robust peak set, defined as being present in both replicates using the bedtools intersect function, was used. Regions of p53 occupancy were determined using the Macs tool (version 1.4) with a p-value cutoff of 1e-5 (suited to narrower peaks), then robust peaks that were present in at least 2 replicates were identified using the bedtools intersect function.

Data quality

Reads with a Phred quality score
of <15 were removed. Non-unique or duplicate reads were removed using samtools and Picard tools
(v1.98) respectively. Regions of BRD4 occupancy were determined using the SICER algorithm with a
redundancy threshold of 1, a window size of 200, a fragment size of 150, an effective genome fraction of
0.75, a gap size of 200 and an FDR corrected p-value of 0.01 (suited to broader BRD4 peaks). To reduce
experimental bias, a robust peak set, defined as being present in both replicates using the bedtools
intersect function, was used. Regions of p53 occupancy were determined using the Macs tool (version 1.4) with a p-value cutoff of 1e-5 (suited to narrower peaks), then robust peaks that were present in at least 2 replicates were identified using the bedtools intersect function.

Software

Sequenced single-end ChIP-seq reads were trimmed using trim-galore (version 3.0) and aligned to the hg19 build of the human genome using Bowtie2 alignment software. Reads with a Phred quality score of <15 were removed. Non-unique or duplicate reads were removed using samtools and Picard tools (v1.98) respectively. Regions of BRD4 occupancy were determined using the SICER algorithm with a redundancy threshold of 1, a window size of 200, a fragment size of 150, an effective genome fraction of 0.75, a gap size of 200 and an FDR corrected p-value of 0.01 (suited to broader BRD4 peaks). To reduce experimental bias, a robust peak set, defined as being present in both replicates using the bedtools intersect function, was used. Regions of p53 occupancy were determined using the Macs tool (version 1.4) with a p-value cutoff of 1e-5 (suited to narrower peaks), then robust peaks that were present in at least 2 replicates were identified using the bedtools intersect function. BRD4 and p53 peaks were visualized using either the UCSC web interface or the WashU browser.

# Flow Cytometry

## Plots

Confirm that:

☐ The axis labels state the marker and fluorochrome used (e.g. CD4-FITC).

☐ The axis scales are clearly visible. Include numbers along axes only for bottom left plot of group (a 'group' is an analysis of identical markers).

☐ All plots are contour plots with outliers or pseudocolor plots.

☐ A numerical value for number of cells or percentage (with statistics) is provided.

## Methodology

Sample preparation

Suspension cells needed minimal preparation for flow sort beyond initial isolation from animals (primary cells), or washes out of a plate (tissue culture).

Instrument

BD Fortessa

| | |
|---|---|
| Software | BD FACSDiva, ModFit |
| Cell population abundance | Relative Cell population was determined with pre-detemined bio-markers depending on the experiment (e.g. CD4+/CD8+). |
| Gating strategy | Gating was designed using the BD Software for optimal efficiency. |

☐ Tick this box to confirm that a figure exemplifying the gating strategy is provided in the Supplementary Information.

# Magnetic resonance imaging

## Experimental design

| | |
|---|---|
| Design type | *Indicate task or resting state; event-related or block design.* |
| Design specifications | *Specify the number of blocks, trials or experimental units per session and/or subject, and specify the length of each trial or block (if trials are blocked) and interval between trials.* |
| Behavioral performance measures | *State number and/or type of variables recorded (e.g. correct button press, response time) and what statistics were used to establish that the subjects were performing the task as expected (e.g. mean, range, and/or standard deviation across subjects).* |

## Acquisition

| | |
|---|---|
| Imaging type(s) | *Specify: functional, structural, diffusion, perfusion.* |
| Field strength | *Specify in Tesla* |
| Sequence & imaging parameters | *Specify the pulse sequence type (gradient echo, spin echo, etc.), imaging type (EPI, spiral, etc.), field of view, matrix size, slice thickness, orientation and TE/TR/flip angle.* |
| Area of acquisition | *State whether a whole brain scan was used OR define the area of acquisition, describing how the region was determined.* |

Diffusion MRI ☐ Used ☐ Not used

## Preprocessing

| | |
|---|---|
| Preprocessing software | *Provide detail on software version and revision number and on specific parameters (model/functions, brain extraction, segmentation, smoothing kernel size, etc.).* |
| Normalization | *If data were normalized/standardized, describe the approach(es): specify linear or non-linear and define image types used for transformation OR indicate that data were not normalized and explain rationale for lack of normalization.* |
| Normalization template | *Describe the template used for normalization/transformation, specifying subject space or group standardized space (e.g. original Talairach, MNI305, ICBM152) OR indicate that the data were not normalized.* |
| Noise and artifact removal | *Describe your procedure(s) for artifact and structured noise removal, specifying motion parameters, tissue signals and physiological signals (heart rate, respiration).* |
| Volume censoring | *Define your software and/or method and criteria for volume censoring, and state the extent of such censoring.* |

## Statistical modeling & inference

| | |
|---|---|
| Model type and settings | *Specify type (mass univariate, multivariate, RSA, predictive, etc.) and describe essential details of the model at the first and second levels (e.g. fixed, random or mixed effects; drift or auto-correlation).* |
| Effect(s) tested | *Define precise effect in terms of the task or stimulus conditions instead of psychological concepts and indicate whether ANOVA or factorial designs were used.* |

Specify type of analysis: ☐ Whole brain ☐ ROI-based ☐ Both

| | |
|---|---|
| Statistic type for inference (See Eklund et al. 2016) | *Specify voxel-wise or cluster-wise and report all relevant parameters for cluster-wise methods.* |
| Correction | *Describe the type of correction and how it is obtained for multiple comparisons (e.g. FWE, FDR, permutation or Monte Carlo).* |

## Models & analysis

n/a | Involved in the study
☐ ☐ Functional and/or effective connectivity
☐ ☐ Graph analysis
☐ ☐ Multivariate modeling or predictive analysis

| | |
|---|---|
| Functional and/or effective connectivity | *Report the measures of dependence used and the model details (e.g. Pearson correlation, partial correlation, mutual information).* |
| Graph analysis | *Report the dependent variable and connectivity measure, specifying weighted graph or binarized graph, subject- or group-level, and the global and/or node summaries used (e.g. clustering coefficient, efficiency, etc.).* |
| Multivariate modeling and predictive analysis | *Specify independent variables, features extraction and dimension reduction, model, training and evaluation metrics.* |
